# A systematic review of task- shifting for HIV treatment and care in Africa

**DOI:** 10.1186/1478-4491-8-8

**Published:** 2010-03-31

**Authors:** Mike Callaghan, Nathan Ford, Helen Schneider

**Affiliations:** 1 Department of Anthropology, University of Toronto, Canada; 2 Médecins Sans Frontières, Cape Town, South Africa; 3 Centre for Infectious Disease Epidemiology and Research, University of Cape Town, South Africa

## Abstract

**Background:**

Shortages of human resources for health (HRH) have severely hampered the rollout of antiretroviral therapy (ART) in sub-Saharan Africa. Current rollout models are hospital- and physician-intensive. Task shifting, or delegating tasks performed by physicians to staff with lower-level qualifications, is considered a means of expanding rollout in resource-poor or HRH-limited settings.

**Methods:**

We conducted a systematic literature review. Medline, the Cochrane library, the Social Science Citation Index, and the South African National Health Research Database were searched with the following terms: task shift*, balance of care, non-physician clinicians, substitute health care worker, community care givers, primary healthcare teams, cadres, and nurs* HIV. We mined bibliographies and corresponded with authors for further results. Grey literature was searched online, and conference proceedings searched for abstracts.

**Results:**

We found 2960 articles, of which 84 were included in the core review. 51 reported outcomes, including research from 10 countries in sub-Saharan Africa. The most common intervention studied was the delegation of tasks (especially initiating and monitoring HAART) from doctors to nurses and other non-physician clinicians. Five studies showed increased access to HAART through expanded clinical capacity; two concluded task shifting is cost effective; 9 showed staff equal or better quality of care; studies on non-physician clinician agreement with physician decisions was mixed, with the majority showing good agreement.

**Conclusions:**

Task shifting is an effective strategy for addressing shortages of HRH in HIV treatment and care. Task shifting offers high-quality, cost-effective care to more patients than a physician-centered model. The main challenges to implementation include adequate and sustainable training, support and pay for staff in new roles, the integration of new members into healthcare teams, and the compliance of regulatory bodies. Task shifting should be considered for careful implementation where HRH shortages threaten rollout programmes.

## Introduction

Sub-Saharan Africa suffers from the world's most pronounced crisis in human resources for health: 36 of the 57 countries that now face health worker shortages are in Africa [1]. These shortages intensify--and are intensified by--the HIV/AIDS pandemic. Much interest has recently been paid to how to streamline HIV care, both to offer high-quality care to patients and expand access to care. One response to this shortage has been the reassignment of clinical roles by shifting tasks to different cadres of health workers: nurses may become involved in prescribing drugs, lay counsellors involved in testing, new cadres may be introduced to perform specific tasks, and patients may be engaged to take over some elements of their own care. The objective is a streamlined, rationalized chain of care that relieves pressure on each worker involved while maintaining quality standards for patients and increasing access to interventions.

Task shifting is not new. In 19th century France, *Officiers de Santé *[2] were an officially recognized and commonly used class of non-physician health care worker, while in China, so-called barefoot doctors were widely deployed across the country in the mid-20th century [3]. In Africa, non-physician clinicians have long been trained across the continent to fill various roles [4-6]. Systematic reviews from various areas of health care provision support the general conclusion that good health outcomes can be achieved by task shifting to nurses [7] and lay or community health workers [8-10].

The potential for task shifting in HIV care was elaborated by the World Health Organization's 2004 publication of Integrated Management of Adult and Adolescent Illness guidelines, which recommended that nurses and clinical aids be trained to provide primary care for HIV [11]. In 2008, this potential was expanded and formalized by joint WHO/UNAIDS/PEPFAR guidelines for the implementation of task shifting [12] as an immediate way to address staff shortages while delivering good quality care. However, the rapidly emerging evidence from sub-Saharan Africa, where task shifting is seen as most relevant, has not been systematically reviewed. Such analysis is important, since task shifting has been the subject of some debate. Critics have argued that task shifting has become a "bandwagon" that is uncritically championed at the expense of existing health cadres, whose low pay and poor working conditions drive high attrition [13]. Several commentators have noted that even though this approach may be able to provide increased quality care for HIV-positive patients, task shifting should not be a substitute for investments in health care systems more generally [14-17], and that even the best staffing models will be inadequate in areas with an absolute shortage of all levels of staff [18]. Concern has also been expressed that shifting additional HIV tasks to lower cadres could risk competing with other service priorities [19,20], particularly given the overall shortage of nurses [21]. In some areas, community health workers already stand in when nurses when are unavailable [22,23].

These concerns underscore the need for careful, critical analysis, particularly where task shifting policies rewrite the job descriptions of some cadres. If task shifting is already widespread in practice, if not in policy, the process should be formalized and rationalized for the long term. This includes ensuring staff competencies and adequate working conditions [24]. This perspective takes for granted the unavoidable necessity of task shifting, and focuses on the need for a timely and logical policy response.

## Methods

We developed a search strategy combining the following search terms: "task shift*" AND "balance of care OR non-physician clinician OR substitute health worker OR community care giver OR primary health care team OR cadres OR nurs*" AND "HIV". Using these terms, we searched the following databases from inception to May 2009: Medline via PubMed, Social Science Citation Index, the South African National Health Research Database, and all the Cochrane Library. The abstract databases of all International AIDS Society Conferences (up to Cape Town, July 2009), all Conferences on Retroviruses and Opportunistic Infections (up to Montreal, February 2009), and all HIV/AIDS Implementers Meetings (up to Windhoek, 2009) were searched. This search was complemented by reviewing the bibliographies of relevant papers and grey literature review, and by personal communication with researchers in the field.

Our review included all articles that detailed approaches to task shifting for the delivery of HIV care in Africa. Abstracts were initially screened by one reviewer (MC) and agreement for final inclusion was sought with other authors (HS, NF).

Although the search methodology was systematic, the paucity and heterogeneity of the results prevent the drawing of systematic conclusions on any particular task shifting practice. We therefore subsequently organized the findings within the context of current debates about task shifting as policy and practice according to five main themes: efficiency; access; quality of care; health outcomes; and team dynamics.

## Results

Our initial search yielded 2960 articles of which 84 were included in the core review. These included articles reporting outcomes (51), review articles (15), opinion pieces and position papers (12), papers elaborating theories and models (13), and policy analysis studies (6). Of those that reported outcomes, 25 were original articles (Table 1); the rest were supplementary presentations of the same study or programme.

**Table 1:** Characteristics and outcomes of studies on the impact of task-shfting in HIV/AIDS care

Study	Setting	Study design	Study size	Intervention	Outcomes
Apondi et al, 2007 [65]; Tugume et al 2009 [66].	Uganda (rural)	Cohort	2522	'Field officers' provide home-based ART	Cumulative outcomes at 4 years showed excellent adherence (96.8% were > 95% adherent) and < 1% defaulting. Social improvements: reduced stigma, stronger family and community relationships

Arem et al, 2009 [69].	Uganda (rural)	Qualitative Survey	---	Peer adherence supporters	Peer health workers successfully understood ART regimens and physical danger signs; 97% of clinic staff reported that peer health workers improved patient outcomes.

Bedelu et al, 2007 [40].	South Africa (rural)	Cohort	1025	Decentralized, nurse-initiated ART	Task-shifted, decentralised care increases access and is more acceptable to patients loss-to-follow-up was clinics 2% at clinics compared to 19% at hospital for comparable virological and immunological outcomes.

Bolton-Moore et al, 2007 [50]	Zambia (urban)	Cohort (paediatric)	2938	Nurse- and clinical officer-initiated paediatric ART	Decentralization allows for dramatically scaled-up rollout; cumulative 3-year mortality (8.3%) and defaulting (5.4%) comparable to other programmes.

Chang et al, 2008 [74]	Uganda (rural)	Cohort	360	Patients trained as 'peer health workers' to monitor ART adherence by mobile phone	Extremely cost effective. 72% retention and 86% virological suppression at 2 years

Chiambe et al, 2009 [42].	Kenya (urban and rural)	Cohort	39,900	Lay health care workers supporting basic clinic tasks and adherence counselling	Enrollment increased from 1,176 to 39,900 patients within 3 years

Chung et al, 2008 [25]	Rwanda (rural)	Modelling	3194	Nurse-initiated ART	Substantial time savings: nurse-initiated ART reduces physician HIV-related workload by 78%, saving up to 56 hours physician time/month.

Cohen et al, 2009 [55].	Lesotho (rural)	Cohort	4,347	Nurse-initiated ART	Favourable outcomes at 12 months among adults (9.3% mortality, 2.5% defaulting) and children (5% mortality, 2% defaulting)

Gimbel-Sherr et al 2008 [48].	Mozambique	Cohort	6,006	ART initiated by mid-level workers (2.5 years training) vs doctors	Patients seen by NPCs (69.4% of cohort) were 44% less likely to be lost to follow up; no difference in mortality

Jaffar et al, 2009 [59].	Uganda (rural)	RCT	859	Home vs clinic-based ART delivery	Similar outcomes of mortality and viral suppression in home-based and faculty-based ART

Koenig et al 2004 [35].	Haiti (rural)	Cohort	2300	Decentralized, CHW-monitored ART	Approach increases access, reduces defaulting, and delays resistance to first-line medication

McGuire et al, 2008 [29].	Malawi (rural)	Cohort	1676	Nurses/medical assistants initiating and managing ART	More rapid time to initiation (21.5 days for nurses/medical assistants vs 35 days for clinical officers); no difference in outcomes and retention rates

Sanjana et al, 2009 [73].	Zambia	Cross-sectional survey	---	Assessment of record-keeping errors among lay vs health care workers	Error rate for lay counsellors was less (6.44/1,000 field) than health care workers (16.81/1,000 fields)

Shulman et al, 2009 [50].	Malawi (rural)	Cohort	---	Lay workers trained as pharmacist assistants	Expanded pharmacy capacity (500 prescriptions per day) and reduced errors (30% to 5%)

Shumbusho et al, 2008 [47].	Rwanda (rural)	Concordance study	---	Nurses trained in ART initiation	Discordance between eligibility and initiation < 1% (n = 343)

Shumbusho 2008 [47].	Rwanda (rural)	Cohort	3194	Nurse-initiated ART	Mortality at defaulting < 5% at 12 months.

Tweya et al, 2008 [64].	Malawi (rural)	Cohort	1,617	Lay-workers to pre-screen for adult ART eligibility	Symptom screening checklist had high sensitivity (91.8%) but low specificity (28%)

Tootla et al 2007 [53].	South Africa (urban)	Cohort	2,084	Nurse/pharmacist managed ART	75% of clients had undetectable viral load at 12 months

Torpey et al 2008 [27].	Zambia	Cohort (quantitative and qualitative analysis)	500	Lay-workers used as 'adherence supporters'	Lay adherence supporters reduced loss-to-follow-up from 15% to 0%; reduced wait times

Udegboka et al, 2009 [28].	Nigeria	Cohort	---	Nurse ART treatment and peer support	Task shifting reduced waiting times by 4 hours

Van Rie et al 2009 [46].	DRC (urban)	Blinded concordance study	339	Nurse vs doctor decisions to initiate ART	95% agreement

Van Griensven et al, 2008 [57].	Rwanda (urban)	Cohort	315	Nurse-initiated and monitored paediatric ART	84% retention and 83% virological suppression at 2 years

Van Griensven et al, 2009 [58].	Rwanda (urban)	Cohort	435	Nurse-initiated and monitored Adult ART	0.3% attrition and 8.5% mortality at 1 year

Wood et al, 2009 [45].	South Africa (urban)	RCT	812	Doctor vs nurse-initiated ART	Non-inferiority according to virological failure, toxicity, adherence, and mortality.

Zachariah et al, 2007 [62].	Malawi (rural)	Cohort	1634	Community support vs no support	26% increase in survival; 98% reduction in loss to follow up.

### Efficiency

We found evidence that task shifting increases programme efficiency. Several studies have quantified time saved by implementing task shifting on the assumption that delegating tasks gives senior clinical staff more time to deal with complicated patients. Time savings are an important outcome for HIV care and could help in addressing bottlenecks in treatment. Authors of a large study in Rwanda assessed time savings from nurse-initiated and monitored antiretroviral therapy (ART), and concluded that such task shifting at the national level would result in a 183% increase in doctor capacity for non-HIV related tasks [25,26]. Reductions in waiting times and loss-to-follow-up have also been observed in task shifted HIV care models [27-30].

Doctor salaries can be the largest cost of running an antiretroviral clinic. One South African study found that doctor salaries constituted roughly 42% of all clinic costs, including utilities and supplies [31]. Reducing dependence on doctors for ART could reduce clinic operating costs, or increase patient load for the same cost. A study comparing total average annual clinic-level cost per ART patient in Uganda and South Africa found that mean costs were almost a third less in the former ($US331 vs $US892) and concluded that task-shifting may have helped to reduce clinic costs and improve overall efficiency [32].

### Access

Efficiencies make possible increased access and affordability. Several studies have also reported an increase in access to counselling and testing through task shifting and the up-training of clinic staff [33-37]. In Botswana, the training of nurses to prescribe and dispense medication increased uptake of antiretroviral therapy, with nearly 20,000 patients receiving treatment at rural clinics as of December 2007 [38]. In Zambia, intensive training in a task shifted model of ART rollout was able to expand treatment access substantially without compromising quality of care [39]. In Lusikisiki, South Africa, district-wide access to ART was achieved within 2 years with a task-shifted model of care [40]. Similar scale up has been reported in Mozambique [41], Kenya [42], and Swaziland [43]. Finally, a costing study from Malawi found that district-wide access to ART using a non-physician model of care was achieved for an additional $2.5 per capita, well within the estimated minimal basic health package costs (WHO) [44].

### Quality of care

Provider performance is a crucial indicator, since lower-level cadres who require constant supervision, or who under-refer or over-refer patients, will save neither time nor money, nor improve the health of their patients. Several studies have evaluated task shifting against a gold standard of care.

We know of only one randomized controlled trial that has assessed the effectiveness of task-shifting for HAART delivery in sub-Saharan Africa. That study found that nurse-managed ART was non-inferior to doctor-managed ART in urban clinics in Johannesburg and Cape Town, South Africa: both treatment arms had similar outcomes of viral suppression, adherence, toxicity and death [45]. A study done in the Democratic Republic of Congo looked at concordance between doctor and nurse decisions to initiate ART and found 95% agreement on ART initiation [46]. Similarly in Rwanda, nurses accurately determined ART eligibility for more than 99% of patients [47]. In Mozambique, patients seen by mid-level workers (with 2.5 years training) were almost 30% more likely to have CD4 counts done at 6 months post ART initiation than those seen by doctors, and were 44% less likely to be lost to follow-up. There were no significant differences in mortality, CD4 counts done at 12 months, or adherence rates [48]. Finally, a study from Malawi found that the training of lay workers as pharmacy assistants reduced prescribing errors by 25% by unburdening the system [49].

### Health outcomes

Several studies have assessed patient health outcomes in HIV services where tasks have been shifted to nurses and lay workers, against internationally accepted standards. A study of nurse-initiated and managed paediatric ART in Zambia--the largest-ever developing-world study of its kind--showed good clinical outcomes [50]. Similarly, a study of a primarily nurse-driven ART program in Kampala, Uganda, reported very good clinical outcomes after 2 years [51]. In each of these examples, the high level of performance of task shifted workers has occurred in a context of in-depth training and ongoing support. The need for ongoing training was highlighted by a study in Mozambique where expert clinicians oversaw the work of mid-level providers and found errors in antiretroviral management in over 40% of cases; errors were associated with duration since pre-service training [52].

A decentralized programme in rural South Africa involved mainstreaming uncomplicated HIV care to lower-level cadres (specifically, nurses and adherence counsellors) in clinics [40]. In a cohort study of 1025 patients, loss-to-follow-up at the decentralized clinics was 2.2%, compared with 19.3% at the relatively centralised hospital, and patients with CD4 > 200 was 87.1% compared with 14.2%. Other programmes in South Africa have reported similarly good outcomes for patients managed by non-physician health workers [53]. Nurse-managed programmes in Lesotho [54,55] and Rwanda [56-58] have also reported highly satisfactory outcomes in terms of mortality and retention-in-care for both adults and children.

Home-based care, treatment support, and other extra-clinical services provided by lay health workers have been shown to be effective in sub-Saharan Africa. A randomized trial in Uganda [59] comparing home-based and facility-based care also found similar rates of viral load suppression, failure and mortality. A community-based program offering home-based ART through lay providers in Uganda achieved excellent outcomes without recourse to regular clinic visits [60]. Adherence to antiretroviral therapy improved after the introduction of lay counsellors and field officers [60,61], with a study from Malawi showing that patients who were offered community support had significantly better survival and retention-in-care rates compared with patients who did not receive such support [61]. In one Malawian study [62], however, community health workers did a worse job of identifying eligible patients for ART than did clinicians. These findings point to the limits to which tasks can be shifted, and underline the need to address the question of what tasks can be delegated, and to whom.

Non-medical patient outcomes have also been measured in task shifted models of care. In Uganda, the implementation of home-based ART through community health workers is associated with positive social outcomes, including an increase in social and family support and strengthened relationships [63-66].

### Team dynamics

The process of task shifting can influence the social dynamics within clinics. An ethnographic study of a task shifted ART scale-up program in Cameroon [67] found a pervasive tension between nurses and community health workers, and ambiguity around the definitions of roles and hierarchies within the clinic. It concluded that task shifting policies must anticipate this problem and clearly delineate processes and responsibilities for existing and newly-created health cadres.

One recent South African study [68] suggested that task shifting leads not only to higher job satisfaction among staff, but to lower workload and usage of sick leave. The same study, however, reported higher staff turnover and poorer physical state of premises at task-shifted clinics. A qualitative survey done in rural Uganda found that almost all clinic staff interviewed (97%; n = 37) strongly agreed or agreed that peer health workers improved the care of patients, and 86% strongly agreed or agreed that peer health workers had made their own jobs easier [69]. In a structured survey conducted among 62 national or provincial managers and HIV clinic staff in Mozambique, respondents indicated that non-physician clinicians should initiate ART for adults (100%), pregnant women (95%), and patients with tuberculosis (83%) [70]. In an evaluation of a programme in Uganda and Zambia where lay counsellors provided basic triage, intensive adherence support and assistance in the provision of ART, their performance was rated as good or very good by 97% of health providers who were interviewed (n = 42); acceptability was also 97% [71].

The importance of ongoing training has been highlighted by qualitative interviews. Community health workers in South Africa [72] report a desire for better training and supervision to meet the formidable challenges posed by the synergy of HIV, tuberculosis and poverty. Similarly, a study done in Zambia found that additional training needs were identified by almost 85% of lay counsellors [73].

Finally, task shifting is recognized as a valuable way to increase patient involvement in care [74]. People living with HIV/AIDS represent a largely untapped pool of treatment supporters, which will continue to grow apace with prevalence. These people are also more likely to remain in their communities than more mobile higher-cadre health workers [75]. Their involvement as active participants in health care delivery will require the negotiation of new power dynamics between patients and care givers and training and supervision where appropriate.

## Assessment of methodological quality of studies

We undertook an assessment of methodological quality for the original studies included in this review (Additional File 1). The criteria related to quality included: sampling, methodology (comparative design or not, including randomization), use of objective outcomes, and discussion on sources of bias and generalizeability of findings. Of the 25 original studies included in this review, 11 included a comparative approach; for 2 studies randomization was done. Most studies (21) used objective outcome measures. Twelve studies were published as fully peer reviewed articles (the rest appeared as conference abstracts), allowing for a more complete assessment. Among these, all employed an appropriate statistical analysis, but only half (6) discussed potential sources of bias. The majority (11) included discussion about the generalizability of findings.

## Discussion and Conclusion

The challenges facing Africa's health care system in responding to the human resource crisis urgently require policies and practices based on robust, policy-relevant evidence [76]. Although formal cost effectiveness studies have not been done, the available evidence for task shifting in HIV care supports the conclusion that it is both effective and economical [77]. Non-physician health care workers are able, with careful training and supervision, to deliver equal and sometimes better results than doctors; similarly there is now considerable evidence regarding the possibility of shifting tasks from professionals or mid-level workers to lay or community health workers. Perhaps most importantly, task shifting seems to substantially expand access to HIV interventions, even in under-serviced areas.

The studies identified in the literature review are marked by substantial heterogeneity [78,79], and highlight several gaps in current research on task shifting. In particular, more research is needed on how the social dynamics in health care teams may be affected by task-shifting policies, as are broader approaches to assessing the outcomes of certain aspects of task shifting, including the management of HAART by cadres lower than nurses. In this regard, while data emerging from randomized controlled trials are important, this approach is unlikely to be the most appropriate, since such complex studies are unlikely to yield data in time to inform such a rapidly changing environment. Nevertheless, our assessment of methodological quality highlights some considerations for improving the design and analysis of future studies. Another important gap relates to the analysis of professional, regulatory and other barriers to policy change in specific contexts.

This review used a comprehensive search strategy that included multiple databases and grey literature sources. The fact that over half of the studies that comprised the core of this review are not yet published in peer-reviewed journals is both strength and a limitation of this review. The aim of systematic reviews is to assemble data from both published and unpublished sources to minimize publication bias. However, the inclusion of unpublished studies may lead to the reporting of problematic information that would otherwise be noted during peer reviews.

Policies on task shifting must be considered in context. Firstly, decisions of exactly which type of task shifting (involving doctors, nurses, community health workers, or patients) to implement will also have to be made according to each country context where task shifting will involve a different set of politics, professional and social dynamics, and resource and training needs. This will determine, in line with available evidence, which cadres can reliably perform which tasks, where to set performance thresholds, and how to ensure the best fit with existing roles and scopes of practice. The importance of processes surrounding task shifting are a recurring theme in the literature: appropriate integration into staff structures, adequate pay, and ongoing support and supervision, all require careful attention. More broadly, task shifting has to be engaged within broader health system goals of building access, equity and responsiveness; and where task shifting involves the mobilisation of community health workers, to questions of community participation and accountability [80].

There appears to be consensus that task shifting alone will not solve human resources problems in HIV services, or in health care more generally, in areas with substantial staff shortages and failing health systems. Indeed, health care worker shortages remain a major impediment to the scale-up of antiretroviral therapy in sub-Saharan Africa. Nor should task shifting be considered simply as a means of saving money: while it makes for more efficient uses of clinical resources, in contexts of worker shortages task shifting is primarily a means of extending access to quality care to a greater number of people. Ultimately, task shifting may offer cost-effectiveness rather than cost-savings, and will require strong government leadership to ensure an enabling regulatory framework, and adequate training and financing [80].

In conclusion, our literature review finds that task shifting is a viable and rapid response to sub-Saharan Africa's human resources crisis in HIV care. Carefully focused action is needed at this stage, not to determine whether task shifting is possible or effective, but to define the limits of task shifting and determine where it can have the strongest and most sustainable impact.

## Competing interests

The authors declare that they have no competing interests.

## Authors' contributions

MC conducted the primary literature review and drafted the manuscript. HS conceived of the review, participated in its design, and helped to draft the manuscript. NF undertook supplementary literature reviews and contributed to the writing of the manuscript. All authors have read and approved the final manuscript.

## Supplementary Material

Additional file 1 Assessment of methodological quality. Click here for file
