# Supplementary material for: A systematic review of task- shifting for HIV treatment and care in Africa
Source: Hum Resour Health. 2010 Mar 31;8:8. doi: 10.1186/1478-4491-8-8 (PMC2873343; doi:10.1186/1478-4491-8-8)
Supplement: Additional file 1 — Assessment of methodological quality. [file 1478-4491-8-8-S1.DOC]

|  | **Methods** | | | | **Results** | | **Discussion** | | |
| --- | --- | --- | --- | --- | --- | --- | --- | --- | --- |
|  | Sampling/eligibility described | Comparative study design | | | Objective outcome measures | Appropriate Statistical analysis | Sources of bias discussed | Relevance to current knowledge | Generalizeability assessed |
|  |  | Before-after | Comparison group | Randomization |  |  |  |  |  |
| Apondi et al, 2007 [65]; Tugume et al 2009 [66]. | + | + | - | - | - | + | + | + | + |
| Arem et al, 2009 [67]. | - | + |  |  | - | - | - | - | - |
| Bedelu et al, 2007 [40]. | + | - | + | - | + | + | - | + | + |
| Bolton-Moore et al, 2007 [50] | + | - | - | - | + | + | - | + | + |
| Chang et al, 2008 [74] | + | - | - | - | + | + | + | + | + |
| Chiambe et al, 2009 [42]. | + | + | - | - | + | - | - | - | - |
| Chung et al, 2008 [25]; Shumbusho 2008 [47]. | - | - | - | - | + | + | - | - | - |
| Cohen et al, 2009 [55]. | - | - | - | - | + | + | - | + | + |
| Gimbel-Sherr et al 2008 [48]. | + |  | + |  | + | + | - | + | - |
| Jaffar et al, 2009 [59]. | + | - | - | + | + | + | + | + | + |
| Koenig et al 2004 [35]. | - | - | - | - | + | + | - | + | + |
| McGuire et al, 2008 [29]. | - | - | + | - | + | + | - | - | - |
| Sanjana et al, 2009 [73]. | - | - | + | - | + | + | - | - | - |
| Shulman et al, 2009 [49]. | - | - | - | - | + | - | - | + | + |
| Shumbusho et al, 2008 [47]. | - | - | - | - | + | + | - | + | - |
| Tweya et al, 2008 [64]. | - | - | + | - | + | + | - | + | - |
| Tootla et al 2007 [53]. | - | - | - | - | + | + | - | - | - |
| Torpey et al 2008 [27]. | + | + | - | - | + | + | + | + | + |
| Udegboka et al, 2009 [28]. | - | - | - | - | - | - | - | - | - |
| Van Rie et al 2009 [46]. | + | - | + | - | + | + | - | + | + |
| Van Griensven et al, 2008 [57]. | + | - | - | - | + | + | + | + | + |
| Van Griensven et al, 2009  [58]. | + | - | - | - | + | + | + | + | + |
| Wood et al, 2009 [45]. | + | - | - | + | + | + | - | - | - |
| Zachariah et al, 2007 [63]. | + | - | + | - | + | + |  | + | + |

Shaded rows indicate studies published as peer-reviewed articles
